# Gastric Duplication in an Adult Female: A Challenging Diagnosis

**DOI:** 10.7759/cureus.31199

**Published:** 2022-11-07

**Authors:** Ariadni Fouza, Ioannis Gkoutziotis, Savvas Tsaramanidis, Apostolos Kamparoudis

**Affiliations:** 1 5th Surgical Department, Ippokrateio General Hospital, Aristotle University of Thessaloniki, Thessaloniki, GRC

**Keywords:** alimentary epithelium, gastric diverticulum, chronic pyloric stenosis, gastric duplication cyst, gastrointestinal duplications

## Abstract

Gastrointestinal duplications can be found in all parts of the gastrointestinal tract. Duplications of the stomach comprise 2-8% of all duplications and are mostly diagnosed during the first year of life. We present a case of a gastric duplication cyst in a 29-year-old female, presenting with epigastric pain and vomiting. Preoperative diagnosis was assumed to be pyloric stenosis. Intraoperatively, a large mass that was attached to the greater curvature was found. Histopathology results were consistent with gastric duplication cyst.

## Introduction

Gastrointestinal duplications (GDs) can be found in all parts of the gastrointestinal tract. They are more common in the ileum, followed by esophagus, jejunum, colon, and stomach [1]. Duplications of the stomach comprise 2-8% of all duplications [1]. They are usually diagnosed during the first year of birth and more than 70% of the cases involve patients younger than 12 years of age [1,2]. We present a case of a gastric duplication cyst in a 29-year-old female that was successfully treated at our center.

## Case presentation

**Figure 1:**
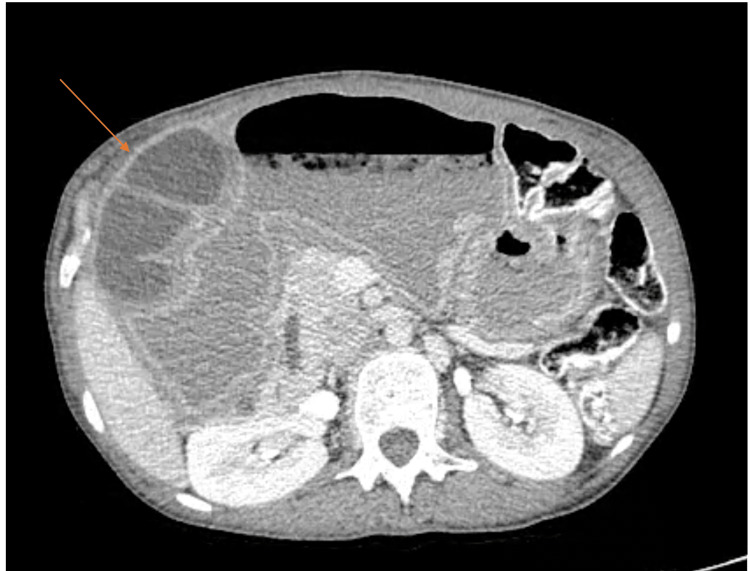
CT scan findings - dilation of the stomach and an associated mass, probably diverticulum, that compressed the stomach.

**Figure 2:**
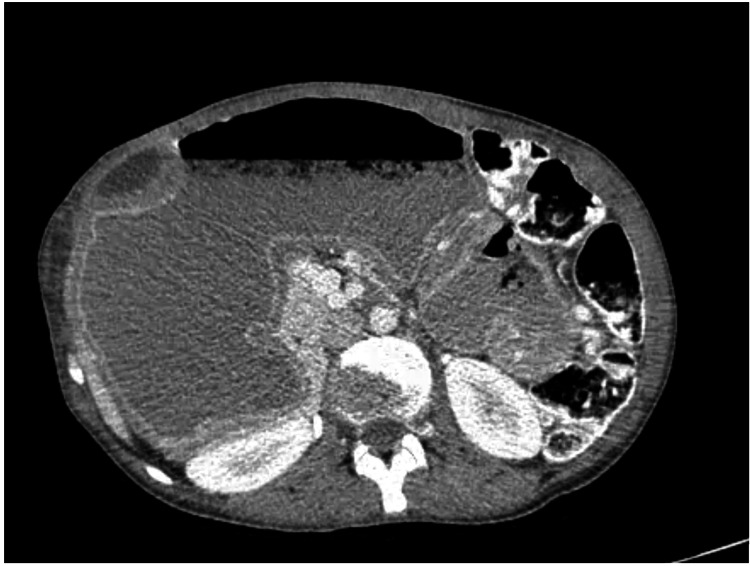
CT scan showed dilation of the stomach and associated compression.

A 29-year-old female of low socioeconomic status presented with a history of epigastric pain associated with vomiting lasting one month. Her medical history included chronic hepatitis B virus (HBV) infection and previous surgery for duodenal atresia during her first year of birth. Furthermore, the patient was remarkably malnourished, reporting periodic symptoms of upper GI obstruction since her childhood. Esophagogastroscopy was performed with failure to pass the pyloric antrum. Thus, no abnormal mucosa was inspected, and the diagnosis of possible pyloric stenosis was assumed. CT scan reported dilation of the stomach and an associated dilated part of jejunum that compressed the stomach (Figures 1, 2).

**Figure 3:**
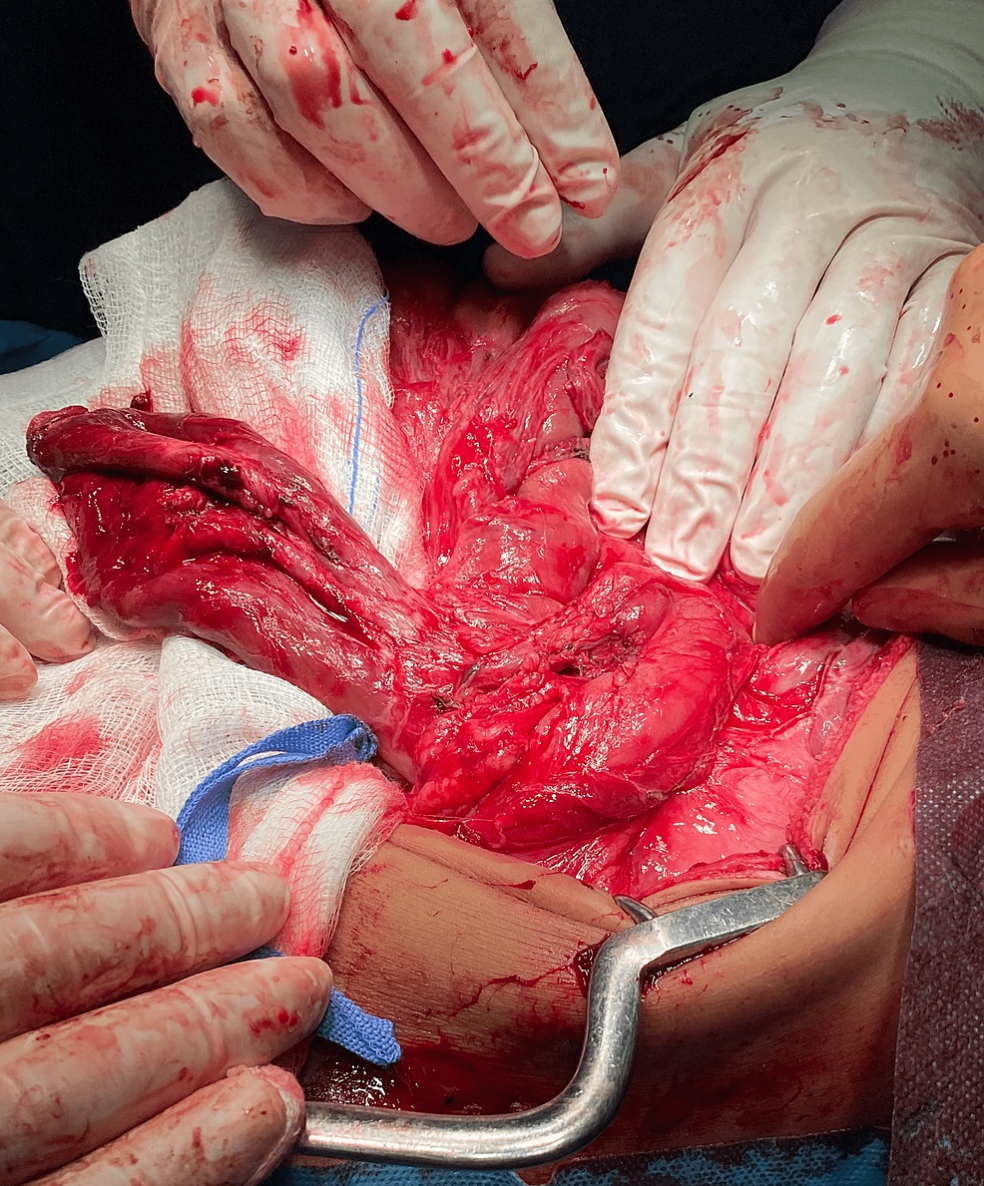
A large cystic mass deriving from the greater curvature with macroscopic characteristics of a diverticulum was found.

**Figure 4:**
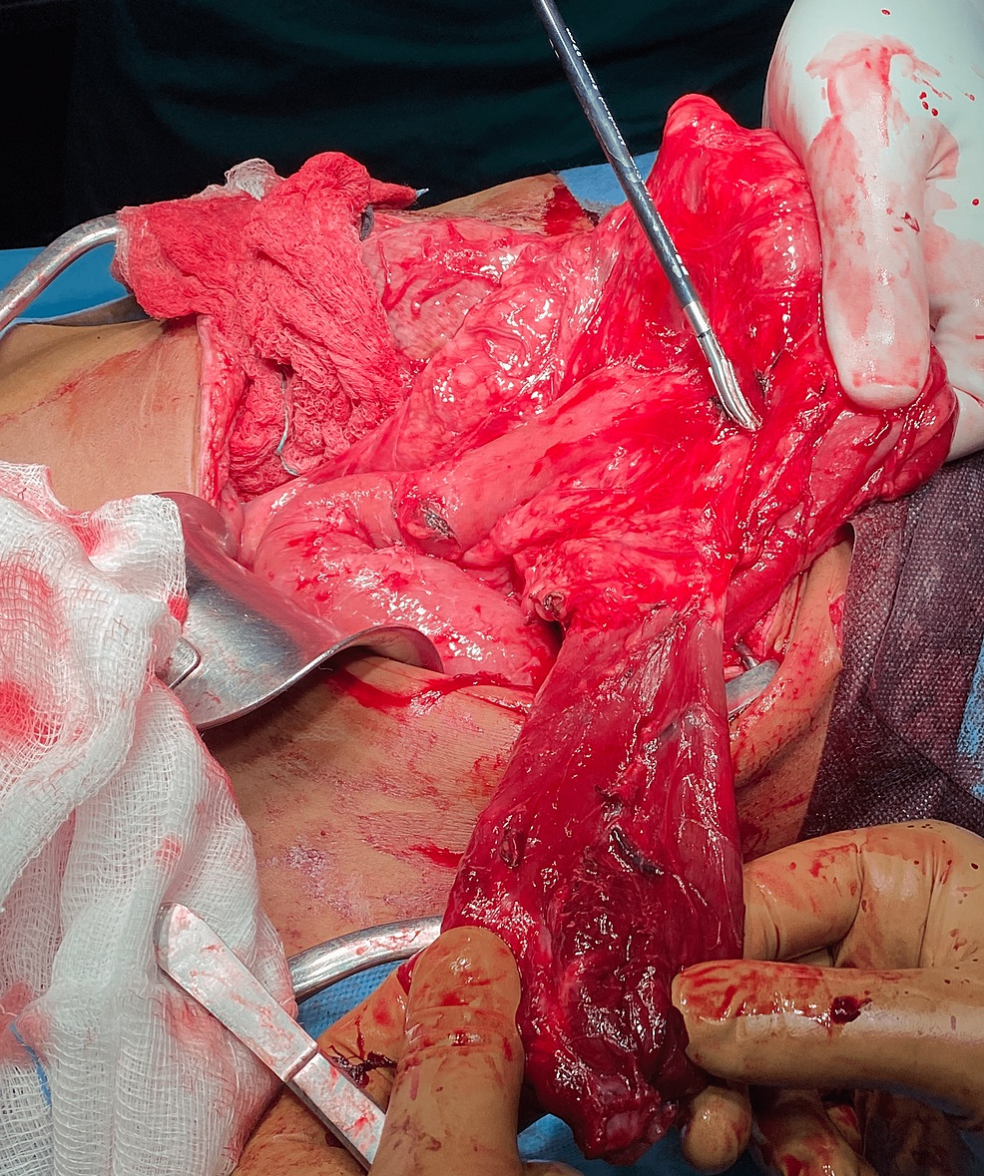
The mass was excised along with part of the stomach.

An exploratory laparoscopy was scheduled, which was converted to an open procedure, due to multiple adhesions. Midline laparotomy was chosen. After extensive adhesiolysis, a large cystic mass arising from the greater curvature with macroscopic characteristics of a diverticulum was found. Wedge resection of the mass was performed, followed by gastrojejunostomy and truncal vagotomy (Figures 3, 4).

According to histologic examination, the specimen had a diameter of 11 cm and length of 8.5 cm and its wall was covered with jejunal epithelium, with parts of chronic inflammation and ulceration. It consisted of mucosa and muscular wall that was not in continuity with the resected part of the stomach. The above-mentioned characteristics are consistent with gastric duplication. The patient had an uneventful postoperative recovery and reports no further symptoms six months postoperatively.

## Discussion

There are two main types of gastric duplications, the cystic and the tubular type. The cystic type comprises 80% of all GDs and they are not in communication with the gastric lumen, whereas the tubular type has some communication with the gastric lumen and is encountered in the remaining 20% of the cases [3]. The diagnosis of GD is based on the following Rowling’s criteria: (a) the wall of the cyst is contiguous with the gastric wall, (b) the cyst is surrounded by smooth muscle, and (c) is lined by alimentary epithelium, which is not always gastric, but can also be colonic, jejunal, or any other type of gut mucosa [3,4]. Other types of cyst epithelium, such as pancreatic or respiratory, have been described [5,6]. Most GDs are found in the greater curvature, but they can also be located in other parts of the stomach [7].

Patients usually describe symptoms of abdominal pain, distention, vomiting, weight loss, palpable epigastric masses, and gastrointestinal bleeding among others [2,3]. GDs are usually associated with other congenital anomalies, such as duplications of other parts of the alimentary tract, ectopic pancreas, esophageal and gastric diverticula, and spinal cord deformities [2,3,8].

Preoperative diagnosis of such duplications is difficult. CT scan is one of the most useful tools and it shows thick-wall cysts with inner layer enhancement [1]. MRI can provide additional information concerning the content of the cysts, but it has not been proven to have diagnostic superiority compared to CT [1,2]. Both methods are reported to be inaccurate in 43-70% of the cases. Ultrasound can also be helpful; GDs are depicted as anechoic cysts [3].

Endoscopic ultrasound is another useful tool in the diagnosis of lesions deriving from the gastric wall and can distinguish their origin as follows: inner echogenic mucosal and outer hypoechoic muscle layers are typical of gastric duplications [9]. Endoscopic ultrasound-guided fine-needle aspiration (EUS-FNA) is also used in order to rule out malignancy, however, its use remains controversial, as elevated levels of carcinoembryonic antigen (CEA) and cancer antigen (CA) 19-9 have been found in samples of benign lesions [9,10]. The differential diagnosis includes gastrointestinal stromal tumors, neuroendocrine tumors, pancreatic heterotopia, pancreatic cystic lesions, and mesenteric cysts among others [9,11-13]. In a retrospective review, which analyzed data from 319 patients, the rate of misdiagnosis was 72.14% [3].

Complications of GDs include infection, bleeding, perforation into the peritoneal cavity, obstruction, and symptomatic compression of adjacent organs [2,7,14]. Malignant transformation has also been reported, with adenocarcinoma being the most frequent type. Therefore surgical treatment of symptomatic cases is recommended [2,14].

## Conclusions

In conclusion, gastric duplication in adult patients is a rare condition that is usually misdiagnosed preoperatively. Due to multiple complications and possible malignant transformation, surgical treatment is indicated. In our case, it is unclear whether the symptoms occurred due to gastric duplication or due to chronic pyloric stenosis. Despite that, excision of the duplication cyst and gastric drainage were necessitated. Vagotomy was preferred to life-long proton pump inhibitors treatment considering the patient’s age and socioeconomic status.
